# Draft Genome Sequence of *Cellulosimicrobium* sp. Strain MM, Isolated from Arsenic-Rich Microbial Mats of a Himalayan Hot Spring

**DOI:** 10.1128/genomeA.01020-14

**Published:** 2014-10-09

**Authors:** Anukriti Sharma, Princy Hira, Mallikarjun Shakarad, Rup Lal

**Affiliations:** Department of Zoology, University of Delhi, Delhi, India

## Abstract

Microbial mats situated at the Manikaran hot springs (>95°C) are characterized by their high arsenic content (140 ppb), qualifying as a stressed niche. Here, we report the annotated draft genome (3.85 Mb) of *Cellulosimicrobium* sp. strain MM, isolated from these microbial mats, consisting of 3,718 coding sequences, with an average % G+C of 74.4%.

## GENOME ANNOUNCEMENT

In an effort to explore the microbial diversity from hot springs located in Manikaran, India, we isolated a thermophilic strain, *Thermus* sp. strain RL, inhabiting water with a surface temperature of >95°C (1). In continuation of this survey, we recently isolated a bacterial strain of the genus *Cellulosimicrobium* from microbial mats (>45°C). The genus *Cellulosimicrobium* is characterized by Gram-positive, rod-shaped, nonmotile chemoorganotrophs (2) and mainly comprises three species: *C. cellulans* (3), *C. funkei* (4), and *C. terreum* (5). Among these, *C. cellulans* and *C. funkei* are reported to be pathogenic (6, 7). The availability of only two genomes of *C. cellulans* J36 and *C. cellulans* LMG 16121 led us to sequence the genome of this strain.

The total genomic DNA of strain MM was isolated using the cetyltrimethylammonium bromide (CTAB) method (8) and was further sequenced using Illumina HiSeq 2000 (2-kb paired-end library) and 454 GS FLX Titanium platforms (2-kb single reads). The sequenced raw data (*n* = 26,536,628) were assembled *de novo* using Velvet 1.2.10 (9), PRICE (10), and minimus2 assembler (11). The assembly produced 299 contigs, for a final assembly size of 3.85 Mbp, with an *N*_50_ of 19,682 bp and a 74.4% G+C content. The NCBI Prokaryotic Genome Annotation Pipeline (http://www.ncbi.nlm.nih.gov/genome/annotation_prok/) and Rapid Annotation using Subsystems Technology (RAST) (12) version 4.0 were used for gene annotation. RAST annotation predicted 3,718 coding sequences and 273 subsystems. ARAGORN (13) predicted 49 genes encoding tRNAs. One copy each of 23S rRNA and 16S rRNA and two copies of 5S rRNA in the draft genome were revealed by RNAmmer (14). Twenty-eight out of 31 protein-coding bacterial phylogenetic markers (15) were retrieved from this draft genome, marking its almost-complete status. 16S rRNA gene-based phylogenetic analysis revealed *C. cellulans*, *Cellulosimicrobium* sp. strain SDE, *C. funkei* strain DBJ, and *Cellulosimicrobium* sp. strain BAB 3237 to be its closest neighbors. Average nucleotide analysis (16) with already-sequenced genomes of *C. cellulans* J36 and *C. cellulans* LMG1621 revealed 88.24% and 98.23% identities, respectively.

*Cellulosimicrobium* strains grow on yeast and fungal cells, which is attributed to their cell wall-degrading enzymes, such as endo-β-1,3-glucanases, proteases, mannanases, and chitinases (17). Genes encoding mannanase were found in strain MM, with *man1*, *man4*, and *man5* being completely reconstructed, with sequence identities of 97.71%, 98.86%, and 98.28%, respectively, with respect to *C. cellulans*. Genes encoding endo-β-1,3-glucanases, endo-β-1,4-xylanases, and chitinases were also retrieved in the draft genome. *C. cellulans* is reported to efficiently utilize baggase (one of the major by-products of the sugar industry) as a carbon source to produce the hydrolytic enzymes xylanase and cellulase (18). These enzymes are further used in breweries, wine making, the paper industry, laundries, and agriculture; hence, in light of the extent and abundance of baggase produced every year, the production of these enzymes is a valuable supplement to the food industry and other industries (18).

### Nucleotide sequence accession number.

The draft genome sequence of *Cellulosimicrobium* sp. MM has been deposited in GenBank under the accession no. JPQW00000000.
